# Carotid Intima-Media Thickness and Cardiometabolic Profile in Turner Syndrome: A Cross-Sectional Study

**DOI:** 10.7759/cureus.61439

**Published:** 2024-05-31

**Authors:** Inês Pais-Cunha, Marisa Pereira, Ana Laura Leite-Almeida, Bárbara Pereira Neto, Sofia Ferreira, Rita Santos Silva, Cintia Castro-Correia

**Affiliations:** 1 Pediatrics, Centro Hospitalar Universitário de São João, Porto, PRT; 2 Pediatric Cardiology, Centro Hospitalar Universitário de São João, Porto, PRT; 3 Pediatric Endocrinology and Diabetology, Centro Hospitalar Universitário de São João, Porto, PRT

**Keywords:** cardiovascular risks, cardiometabolic, turner syndrome, metabolic syndrome, growth hormone

## Abstract

Introduction: Turner syndrome (TS), one of the most common chromosomal abnormalities in females, often results in adult cardiovascular and metabolic complications. Information on pediatric age is scarce. This study aimed to compare the presence of cardiometabolic risk factors in children with TS and healthy controls.

Methods: This is a cross-sectional study comparing patients with TS to age-matched healthy controls, regarding cardiometabolic risk factors including lipid profile, fasting glucose, insulin resistance, body composition, body mass index, blood pressure, and carotid intima-media thickness (cIMT).

Results: We included nine TS patients and nine controls with a median age of 13 years (9-14 years). Three TS patients and three controls were prepubertal. All TS patients received growth hormone treatment (GHT), median treatment of six years (3-10 years); four patients underwent treatment with estradiol. No statistically significant differences were detected between TS patients and controls regarding body mass index (BMI), cholesterol levels, and insulin resistance. cIMT indexed to body surface area showed no significant differences between TS patients and controls (0.37 vs 0.35 mm/m^2^, respectively, p=0.605). TS patients had lower body fat levels (7.2% vs 34.9%, p=0.004). On the other hand, TS patients had higher levels of systolic (z-score 1.04 vs -0.08, p=0.001) and diastolic (z-score 1.08 vs 0.33, p=0.031) blood pressure (BP) and aspartate (AST) and alanine (ALT) aminotransferase levels (26 vs 20 U/L, p=0.008 and 19 vs 14 U/L, p=0.004, respectively).

Conclusion: Patients with TS, all submitted to GHT, had lower body fat levels compared with controls, despite similar BMI. Although we found no differences in cIMT between the two groups, young girls with TS had higher BP and transaminase levels. Early anthropometric, cardiovascular, and analytical monitoring of patients with TS is essential to detect abnormalities and prevent further complications.

## Introduction

Turner syndrome (TS) is a condition that results from a completely or partially missing X chromosome. This is the most common chromosomal anomaly in females, occurring in approximately one in 2000-3000 live female births [1]. Girls and women with TS typically present with short stature and ovarian dysfunction. These patients usually have primary amenorrhea and only 10% evidence of spontaneous menarche [2]. Other characteristic phenotypical findings include a short neck, broad chest, and the presence of Madelung deformity of the forearm and wrist [3].

The overall mortality rate for patients with TS is higher than the general population [4]. This can be explained, in part, by the increased incidence of cardiovascular diseases in these patients [5]. TS patients seem to have altered lipid profiles with lipid accumulation in abdominal viscera, leading to metabolic dysregulation [6]. This dysregulation is associated with several comorbidities, including liver disease which can also be caused by architectural abnormalities [7]. In addition to the impact caused by dyslipidemia and metabolic syndrome, TS has been associated with inherent vascular anomalies, including arterial stiffness and endothelial dysfunction [8,9].

Progressive vasculopathy, hypertension, and coronary heart disease, frequently associated with insulin resistance and altered body composition, are predisposing risk factors for excess morbidity and mortality in adulthood [10-12]. Although these complications have been well established in the adult population, some studies have also shown that changes may appear earlier in life. In fact, increased blood pressure (BP) and arterial stiffness, as well as signs of metabolic disorder have been described in younger patients [13-15]. Nevertheless, there is still a lack of literature focusing on pediatric age, making it challenging to plan appropriate screening and monitoring of this population.

In order to fill this gap, the aim of our study was to evaluate and compare cardiometabolic risk factors including lipid profile, fasting glucose, insulin resistance, body composition, body mass index, blood pressure, and carotid intima-media thickness in a group of pediatric patients with TS and healthy subjects. We also wanted to compare transaminase levels, as markers of liver disease, between children with TS and the general population.

## Materials and methods

This cross-sectional study was carried out in a tertiary hospital, Centro Hospitalar Universitário de São João, from May to June 2023.

Participants

We included all patients with a cytogenic diagnosis of TS between the ages of 7 and 18 years, followed in the Pediatric Endocrinology Unit of our hospital. Since cardiometabolic issues are more likely to be relevant and require intervention in older children, we chose an age cut-off that only included school-aged and adolescent girls. On the other hand, reference values for younger children still need to be well established, which could interfere with our results.

Age and sex-matched controls were healthy children who presented to the hospital for follow-up consultations on minor illnesses such as urinary tract infection or iron-deficiency anemia. We collected blood samples of the control group at the time of the follow-up examination, after the resolution of any acute complaints. Children were excluded if they were on chronic medication or had a history of cardiovascular, endocrine, or metabolic disease. Subjects were invited to participate in the study in person, during their medical consultation, or by phone call to come for an anticipated appointment. We were able to contact all 10 patients with TS that fulfilled the inclusion criteria. One patient declined to participate. The remaining nine patients were included in the study. All patients underwent anthropometrical and BP measurements and collected fasting blood analysis. A carotid artery ultrasound was performed to measure carotid intima-media thickness (vide infra). Past medical and family history were collected from the medical interview as well as the patient's electronic medical records.

The study was approved by the Ethics Committee of Centro Hospitalar Universitário de São João/Faculdade de Medicina da Universidade do Porto and complies with the World Medical Association Declaration of Helsinki regarding the ethical conduct of research involving human subjects (#91/2023). Information regarding the study design and purpose was delivered to caregivers, and signed informed parental consent was obtained from all children.

Anthropometric measurements

Participants were evaluated in underwear and bare feet. Weight was measured to the nearest 0.1 kg using a digital scale (Arlington Heights, IL: Tanita), and standing height was measured to the nearest 0.1 cm using a wall stadiometer (Hamburg, Germany: Seca). Body mass index (BMI) was calculated by dividing weight (kg) by squared height (m^2^). Weight, height, and BMI were transformed into age and sex-specific z-scores based on the Centers for Disease Control and Prevention (CDC) guidelines [16]. Body surface area (BSA) was calculated using the Du Bois method [17]. Body composition was assessed by bioelectric impedance (Arlington Heights, IL: Tanita) and whole-body dual x-ray absorptiometry scans (Bedford, MA: Hologic QDR Discovery 4500W).

Blood pressure

Systolic blood pressure (SBP) and diastolic blood pressure (DBP) were measured by the same investigator (IPC) for all patients at the time of their study visit, after a 10-minute rest. An appropriately sized cuff was placed on the right arm, supported at heart level, and measurements were performed after five minutes of rest in a quiet environment with the child seated with her back and feet in a supported position. The cuff size was chosen so that the bladder width was approximately 40% of the circumference of the upper arm, and the bladder length was chosen to encircle 80-100% of the circumference of the upper arm, midway between the olecranon and the acromion. BP was taken at least twice with an aneroid sphygmomanometer (Erka Vario Desk Model; Bad Tölz, Germany: ERKA) [18]. If the second value was more than 5 mmHg different from the first, continued measurements were made until a stable value was obtained. The final recorded value was the average between the last two measurements. Systolic and diastolic BP are presented as a z-score based on the National High Blood Pressure Education Program Working Group recommendations [19].

Tanner stage

Sexual development evaluation was classified by a doctor (IPC) according to the sexual maturation ratings including breast changes and pubic hair changes (Tanner stages). Breasts were evaluated by inspection and palpation and classified in B1-B5, and pubic hair in P1-P5 [20]. Menarche was self-reported.

Laboratory analysis

In each follow-up visit, an overnight fast venous blood sample was obtained before 11:00 a.m., after applying topical analgesic with lidocaine/prilocaine (EMLA cream). Insulin was measured by electrochemiluminescence immunoassays on the Cobas E411 analyzer (Basel, Switzerland: Roche Holding AG). HbA1c was determined by high-performance liquid ion-exchange chromatography (Bio-Rad VARIANT II; Hercules, CA: Bio-Rad Laboratories, Inc.), glucose was measured using a UV enzymatic assay (hexokinase method), and total cholesterol, HDL-cholesterol (HDL-C), and triglycerides (TG) and transaminase levels were measured using conventional assays, all on a Beckman-Coulter AU5400 (Brea, CA: Beckman Coulter, Inc.) automated clinical chemistry analyzer. LDL-cholesterol (LDL-C) was calculated using the Friedewald formula. The homeostatic model assessment-insulin-resistance (HOMA-IR) was computed as “glucose (mg/dL) x insulin (mU/mL)/405.”

cIMT measurements

All echocardiographic assessments were performed in all patients by a pediatric cardiologist who was blinded to the participants’ clinical data, risk factors, and laboratory test results. A GE Vivid E95 (Chicago, IL: GE Healthcare) ultrasound imaging system, equipped with a high-frequency matrix linear array (5.0-13.0 MHz) vascular ultrasound transducer and a three-lead electrocardiogram, was used for the echocardiographic assessments. The measurements were obtained using the standards recommended by the Association for European Paediatric Cardiology [21].

All participants were examined in the supine position with their necks extended and turned away from the transducer. Both the left and right common carotid arteries were evaluated. For carotid intima-media thickness (cIMT) measurements, longitudinal images of the common carotid artery were obtained.

In the far wall of the common carotid artery, the leading edges of the lumen-intima and media-adventitia interfaces (the "double-line pattern," representing the intima-media complex) were identified. The examinations were saved in Digital Imaging and Communications in Medicine (DICOM) format and exported to a remote computer for measurements. cIMT is indicated by the distance between both leading edges of the lumen-intima and media-adventitia interfaces, measured in the end-diastolic phase. The mean cIMT was calculated for each left and right common carotid artery as the average of three consecutive measurements. All values are presented as absolute and body surface area-corrected indices.

Statistical analysis

Statistical analysis was carried out using SPSS Statistics for Macintosh version 29.0.2.0 (Armonk, NY: IBM Corp). We compared the outcomes of TS patients with age- and sex-matched healthy controls. Categorical variables were presented as frequencies and percentages. The chi-squared test was used to compare categorical variables when there was no more than 20% of the expected values less than 5, when this assumption was not met, we used the Fisher's exact test. Continuous variables were expressed as medians and interquartile ranges (IQR) and compared using the Mann-Whitney U test due to the non-normal distribution of the data. A p-value less than 0.05 was considered statistically significant.

## Results

The anthropometric and baseline characteristics of TS patients and controls are described in Table 1. Median age was 13 (9-14) years in both groups. Three patients in the TS and three subjects in the control group were prepubertal. All TS patients received growth hormone treatment (GHT) with a median duration of six (3-10) years. Among this group, four patients received treatment with transdermal estradiol with a median starting age of 12.9 (12.7-13.2) years. Four patients (44%) in the TS group had a history of congenital heart defects. Three had a bicuspid aortic valve, one had an aortic coarctation, and one had an auriculoventricular septal defect. In the control group, two subjects (22%) had a family history of cardiovascular disease while there was no family history in the TS group (p=0.235).

**Table 1:** Anthropometric and baseline characteristics of TS patients and controls. TS: Turner syndrome

Variables		TS (n=9)	Controls (n=9)	p-Value
Age median, years median (IQR)		13 (9-14)	13 (9-14)	1
Tanner stage 1, n (%)		3 (33)	3 (33)	1
Congenital heart defect, n (%)		4 (44)	0	0.081
Family history of cardiac disease, n (%)		0	2 (22)	0.471
BMI median, z-score median (IQR)		0.4 (-0.7-0.6)	1.3 (0.2-2.3)	0.113
Body fat, % median (IQR)		7.2 (4.9-18.3)	34.9 (24.2-38.9)	0.004
BSA, m^2 ^(median, range)		1.2 (0.9-1.3)	1.5 (1.3-1.7)	0.014
Growth hormone treatment, n (%)		9 (100)	-	-
1.	Previous, n (%)	1 (11)	-	-
2.	Ongoing, n (%)	8 (89)	-	-
3.	Treatment duration, years median (IQR)	6 (3-10)	-	-
Estrogen treatment, n (%)		4 (44)	-	-
1.	Previous, n (%)	1 (11)	-	-
2.	Ongoing, n (%)	3 (33)	-	-
3.	Age of initiation, years median (IQR)	12.9 (12.7-13.2)	-	-

Regarding the anthropometric measurements, there were no statistical differences between the median BMI z-score in the TS and the control group (0.4 vs 1.3, respectively, p=0.113). Median body fat percentage was higher in controls compared to TS girls (34.9 vs 7.2, respectively, p=0.004). On the other hand, TS patients had a lower median BSA when compared to controls (1.2 vs 1.5, respectively, p=0.014).

Concerning the fasting analysis, there were no statistical differences between the median levels of total cholesterol (p=0.796), low-density lipoprotein (LDL) cholesterol (p=0.481), high-density lipoprotein (HDL) cholesterol (p=0.546), triglycerides (p=0.965), and homeostatic model assessment for insulin resistance score (HOMA-IR) (p=0.815). Aspartate (AST) and alanine (ALT) aminotransferase median levels were higher in TS patients (26 vs 20 U/L, p=0.008, and 19 vs 14 U/L, p=0.004, respectively). Median cIMT indexed to BSA showed no significant differences between TS patients and controls (0.37 vs 0.35 mm/m^2^, p=0.605, respectively) (Table 2).

**Table 2:** Fasting serological analytical results and cIMT measurements. *One missing value of HOMA-IR, score median (IQR). TS: Turner syndrome

Variables			TS (n=9)	Controls (n=9)	p-Value
Fasting serological analysis					
1.	Total, mg/dL median (IQR)		145 (137-161)	146 (125-152)	0.796
2.	LDL, mg/dL median (IQR)		85 (67-98)	77 (67-86)	0.481
3.	HDL, mg/dL median (IQR)		54 (47-57)	53 (48-54)	0.546
4.	Triglycerides, mg/dL median, (IQR)		63 (58-69)	60 (47-97)	0.965
5.	HOMA-IR, score median (IQR)*		2.4 (1.2-21.8)	3.1 (1.9-3.9)	0.815
6.	AST, U/L median (IQR)		26 (24-40)	20 (20-25)	0.008
7.	ALT, U/L median (IQR)		19 (17-36)	14 (11-17)	0.004
cIMT					
8.		Absolute values, mm/m^2^ median (IQR)	0.42 (0.37-0.45)	0.52 (0.48-0.57)	<0.001
9.		BSA indexed values, mm/m^2^ median (IQR)	0.37 (0.32-0.45)	0.35 (0.32-0.41)	0.605

**Figure 1:**
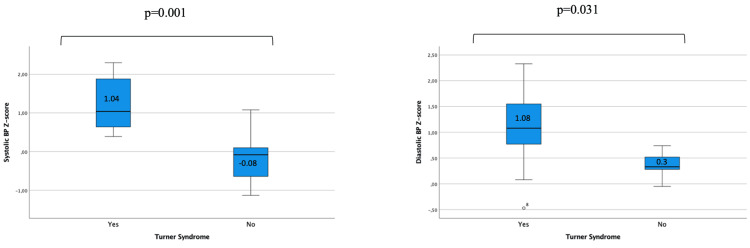
Box plot of BP systolic and diastolic z-score measurements in TS and controls. TS: Turner syndrome

Concerning BP measurements, TS patients had higher levels of median systolic (z-score 1.04 {0.6-2.0} vs -0.08 {-0.8-0.3}, p=0.001, respectively) and diastolic (z-score 1.08 {0.4-1.7} vs 0.33 {0.2-0.6}, p=0.031, respectively) BP z-scores compared to controls (Figure 1).

Within the TS group, there were no differences between patients with and without congenital heart disease regarding median systolic (z-score 0.8 {0.6-1.9} vs 1.5 {0.7-2.0}, respectively, p=0.730) and diastolic (z-score 1.1 {-0.2-2.1} vs 1.1 {0.4-1.7}, respectively, p=1.0) BP.

## Discussion

In our study, we underline the following three major findings: TS patients, treated with GH, showed lower body fat levels, presented with higher systolic and diastolic BP, and did not show differences in cIMT measurements when compared with healthy individuals.

Firstly, TS patients had significantly lower levels of body fat when compared to controls, despite similar lipid profile analysis and similar BMI. We hypothesize that these outcomes may be related to the fact that all of our TS patients were submitted to GHT. GHT is described as promoting anabolism of lean tissue and reducing adiposity, having a positive impact on body composition [22]. Nevertheless, this conversion of adipose tissue to lean body mass does not mean a reduction in weight. Studies in children have shown that there is a positive effect of this therapy on body composition, independent of weight and BMI [23]. In fact, Gnacińska et al. found that young women with TS who had undergone GHT had lower percentages of fat tissue, but not lower BMI, when compared to those who were not given this treatment [24]. Ari et al. compared pediatric TS patients treated with GH to those who did not receive this treatment. Their study revealed that GH-treated patients exhibited, on average, a 15% decrease in adipose tissue compared to untreated patients (p<0.001) [25]. However, it should be noted that although there was no statistical difference in BMI between controls and TS patients, the control group had higher values, which could also be related to their body composition and explain why their body fat levels were higher. These findings in otherwise healthy individuals probably reflect the increasing levels of childhood obesity in Europe, with an estimated prevalence of one in three school-aged children [26].

Secondly, we found increased levels of systolic and diastolic BP in TS girls. Although these levels were within the normal range, this difference may reflect an underlying vasculopathy and future hypertension. In adult women with TS, the prevalence of hypertension can reach 60% [27] and can be the result of renal anomalies, frequent in this population, or maybe idiopathic [28]. Given that hypertension is associated with aortic dilation [29], early routine monitoring is imperative for the identification of hypertension and timely treatment.

Thirdly, we did not find higher cIMT scores in TS children. Increased cIMT is associated with an increased cardiovascular risk, being a precursor of atherosclerotic plaque [30]. This score has been described to be elevated in adults with TS and has been linked to several factors including estrogen deficiency. It appears, in fact, that abnormal cIMT levels are associated with short duration of estrogen treatment in adult women [31].

Nevertheless, similar to our results, other studies on pediatric age also did not find higher cIMT scores in TS children when compared to the general population [32,33]. It should also be noted that, although GHT has been related to cardiovascular events in some cohorts [34]. cIMT levels do not differ significantly between children with or without GHT [35].

Lawson et al. found lower cIMT absolute scores in young TS patients compared to lean and obese controls but showed functional differences with impairment in other measurements such as pulse wave velocity femoral, Young’s elastic modulus, and augmentation index [36]. These differences may precede cIMT structural changes. Although we did not perform functional tests in our patients, given that we found higher BPs despite similar cIMT values in TS patients, we can hypothesize that hemodynamic changes may precede an increase in cIMT measurements in these patients.

Finally, TS girls had higher transaminase levels when compared to controls, although within normal range. These changes may precede adult liver dysfunction, which underlines the importance of routine analyses in this population [37]. Liver involvement in TS includes lesions related to metabolic disorders (steatosis, steatofibrosis, and steatohepatitis) but also to architectural changes due to congenital vascular disorders and even small duct alterations [7]. Although the administration of exogenous estrogen substitution therapy is known to cause liver enzyme abnormalities, estrogen deficiency is also detrimental to liver health. In the case of TS patients, hormone replacement therapy has the potential to normalize abnormal transaminase levels [38]. Current guidelines advise for appropriate initiation of hormone therapy to improve liver function as well as annual monitoring of liver function from the age of 10 onwards [28].

Furthermore, it is also important to note that, although our TS group did not show an increase in HOMA score, given that GHT is associated with hyperglycemia and insulin resistance, it is important to perform routine controls to detect any complications in due time [39].

The strengths of this study include the high participation adherence of our TS cohort (90%) and a study design that enabled us to control the outcome variables. Fasting analyses were performed in the same laboratory and each measurement (anthropometrical, BP, and cIMT) was performed by only one investigator each, reducing interobserver bias. In addition, to our knowledge, this is one of the few studies in the literature focusing on the cardiometabolic profile of pediatric patients with TS, highlighting its novelty.

Nevertheless, this was a cross-sectional study with its inherent limitations. Measurements were only considered at one point in time, and we were not able to investigate cardiovascular changes in the same patient over the years. On the other hand, given that this was a single-center study of a relatively rare condition, we were only able to gather a small cohort of patients and perform a description of our findings, which limits the generalizability of the findings. Given the limited time and resources available for this study, we were not able to increase the control group to a 1:2 ratio, but this may be a way to gain statistical power in future studies. The fact that we chose controls from a hospital population can also constitute a source of bias. Future multi-centric studies with community controls could be of interest to supplement our findings. While acknowledging the limitations of studying a small population, our article provides valuable insight into a relatively understudied population - pediatric patients with TS. Although causality cannot be established with this study design and the associations are not very strong given the sample size, the description of our findings, in addition to the current literature, allows for a careful interpretation of our results. By highlighting aspects of the vascular and cardiometabolic profile of this complex population, our results pave the way for future research and guidance in monitoring and management strategies.

## Conclusions

In conclusion, we found that our cohort of young individuals with TS, treated with GHT, had lower levels of body fat compared to controls, despite similar BMI. Although cIMT levels were not elevated in this group, girls with TS had higher BP and transaminase levels compared to healthy children, which may be related to underlying vasculopathy and future hypertension and liver dysfunction. These results highlight the importance of regular anthropometric, cardiovascular, and analytical monitoring of patients with TS to detect early abnormalities and prevent further complications.
